# Ten simple rules for building an antiracist lab

**DOI:** 10.1371/journal.pcbi.1008210

**Published:** 2020-10-01

**Authors:** V. Bala Chaudhary, Asmeret Asefaw Berhe

**Affiliations:** 1 Department of Environmental Science and Studies, DePaul University, Chicago, Illinois; 2 Department of Life and Environmental Sciences, University of California, Merced, California; Carnegie Mellon University, UNITED STATES

## Abstract

Demographics of the science, technology, engineering, and mathematics (STEM) workforce and student body in the US and Europe continue to show severe underrepresentation of Black, Indigenous, and people of color (BIPOC). Among the documented causes of the persistent lack of diversity in STEM are bias, discrimination, and harassment of members of underrepresented minority groups (URMs). These issues persist due to continued marginalization, power imbalances, and lack of adequate policies against misconduct in academic and other scientific institutions. All scientists can play important roles in reversing this trend by shifting the culture of academic workplaces to intentionally implement equitable and inclusive policies, set norms for acceptable workplace conduct, and provide opportunities for mentorship and networking. As scientists are increasingly acknowledging the lack of racial and ethnic diversity in science, there is a need for clear direction on how to take antiracist action. Here we present 10 rules to help labs develop antiracists policies and action in an effort to promote racial and ethnic diversity, equity, and inclusion in science.

## Introduction

Racial and ethnic diversity in the science, technology, engineering, and mathematics (STEM) workforce remains low, particularly at the PhD level and above [1–3]. The May 2020 murder of George Floyd sparked a global uprising against systemic racism and police brutality against Black people [4]. At the same time, racism faced by Christian Cooper while birding in Central Park in New York City demonstrated the danger that Black scientists face in natural spaces, including during scientific fieldwork and while being #BlackInNature [5]. Days later, Black academics began sharing on social media thousands of harrowing stories accounting the racism they face in academic institutions using the hashtag #BlackintheIvory [6]. On June 8, 2020, Black scientists initiated a global strike to eliminate racism and encouraged colleagues to spend the day reading about antiracism, reflecting on its pervasiveness, and developing antiracism plans of action [7]. These events infused new energy into decades-long efforts working to address racial inequities in STEM [8–10].

Scientists increasingly acknowledge the problematic lack of racial and ethnic representation of Black, Indigenous, and people of color (BIPOC) in science and are in search of clear actionable steps they themselves have the power to immediately enact. Professional scientific organizations, universities, and departments all have a role in developing programs and policies that promote racial and ethnic diversity, equity, and inclusion (DEI). In addition, there are swift actions that research group leaders or primary investigators (PIs) can take to build a lab environment that fosters a racially inclusive environment and ultimately promotes DEI across scientific fields.

Scientists who are beginners to discussions of race, lacking guidance or background knowledge, may adopt unevolved viewpoints or weak policies that unintentionally harm BIPOC [11] or contribute to an erosion of trust among people of different racial or ethnic backgrounds in a lab group. Harmful approaches include engaging in objectifying thought experiments that question the instrumental value of BIPOC in science, confusing race as a biological entity as indicated by human genetic variation instead of a socially constructed concept [12], arguing that the unbiased nature of science and scientists precludes racial biases in scientific workplaces, and hijacking discussions of race with anecdotes from other types of discrimination (e.g., gender-based and/or class-based) without employing an intersectional framework [13]. As scientists of color who actively engage in work to promote racial and ethnic DEI, we have encountered all of these harmful scenarios and more.

Building a lab that is antiracist is very different from building a lab that simply avoids racism. Avoiding racism or stating that one’s lab is “not racist” adopts a neutral stance in a struggle that inherently has no neutrality [14]. As the scholar Ibram X. Kendi writes, “One either allows racial inequities to persevere, as a racist, or confronts racial inequities, as an antiracist. There is no in-between safe space of ‘not racist.’” [15]. We support recent calls to promote the health and well-being of lab members [16] and supportive lab groups that are resilient to outside stressors [17]. But building an antiracist lab goes beyond being kind, treating people equally, or taking a color-blind approach. Being antiracist means developing and supporting antiracist policies through intentional introspection and subsequent action.

**Fig 1:**
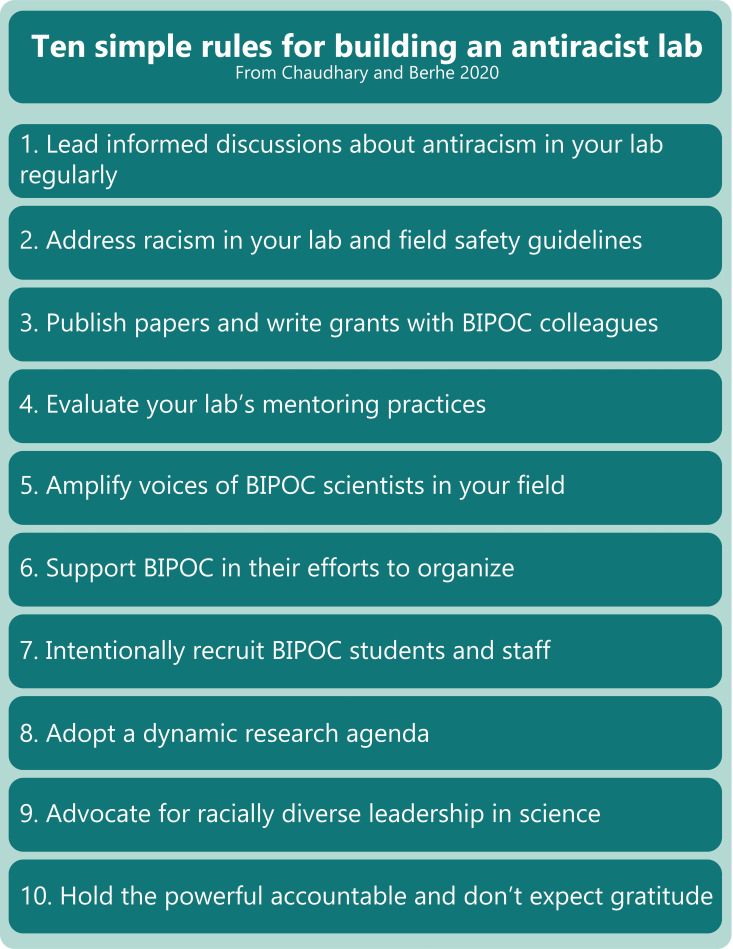
Summary of rules and poster to facilitate antiracist dialogue in science workplaces.

**Fig 2:**
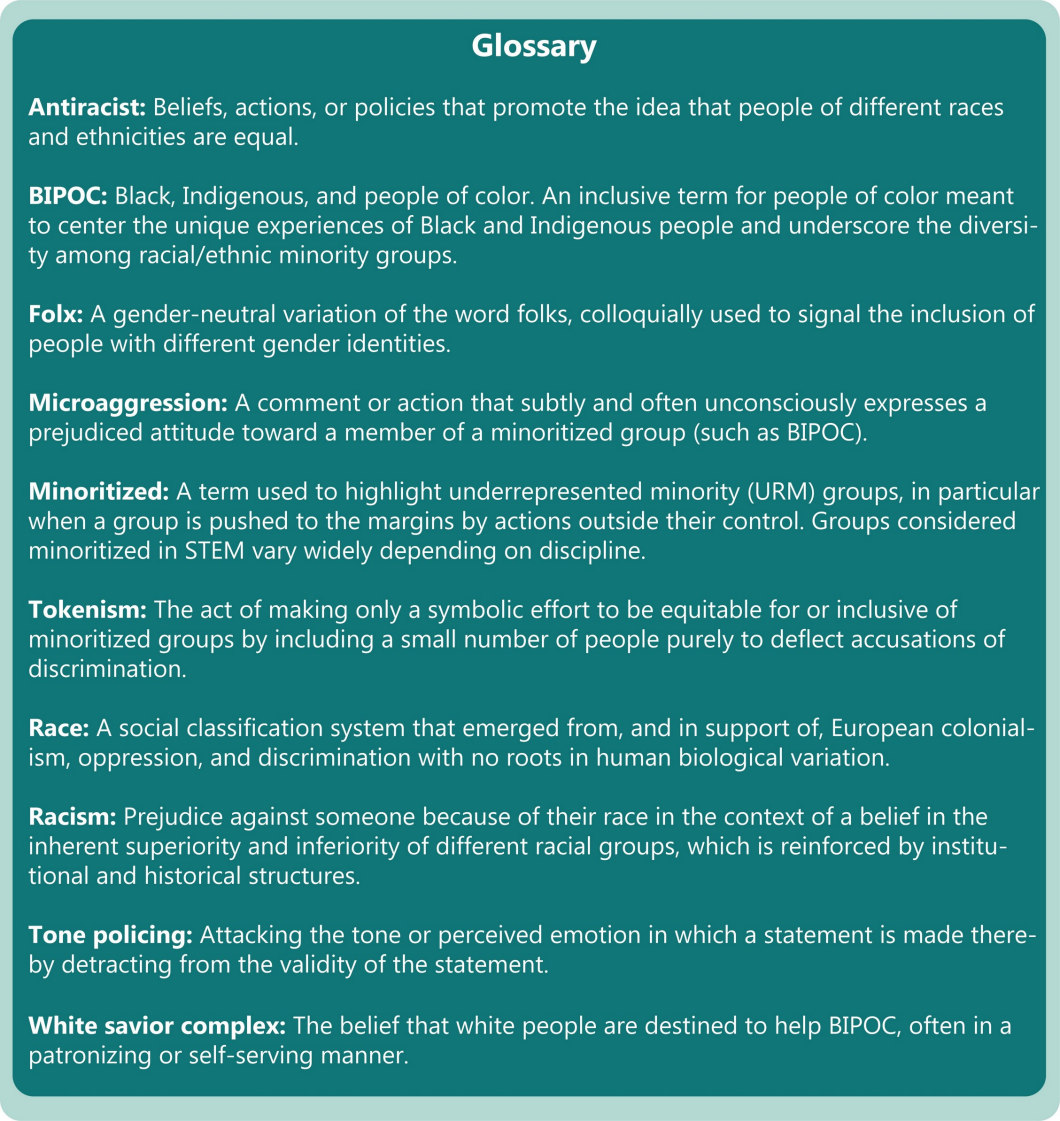
Glossary of terms commonly encountered during antiracism discussions.

Many current and future PIs are looking for clear advice on how to move beyond statements of solidarity and toward concrete achievable antiracist action in their labs. We share these 10 rules (Figs 1 and 2) to contribute to antiracist STEM discourse and help springboard scientists toward immediate achievable action in realms under their control. It is our hope that partaking in such actions will help lead to improved racial and ethnic diversity and inclusion in the lab and successful scientific lives for all.

## Rule 1: Lead informed discussions about antiracism in your lab regularly

Most PIs would be appalled to learn about members of their lab group being victims of racism. Unfortunately, both overt and covert racist incidents (e.g., microaggressions, tokenism, white savior complex, tone policing, etc.) routinely occur in science labs and go unreported. Unreported racism leads to isolation, anxiety, and stress among BIPOC and can ultimately lead to BIPOCs leaving STEM fields. Part of the responsibility of a PI in creating a safe working environment is developing a lab where lab members feel comfortable talking about race and reporting racism, including individual, institutional, or systemic racism in society and especially within academic workplaces [18]. Leading regular discussions on race informed by scholarly readings signals to lab members, BIPOC and white, that racial discrimination is not tolerated and that silence is implicit acceptance of racism [19]. Initiate a preliminary discussion on promoting antiracist STEM with lab members and then revisit the topic each term. Discussions can be structured around journal club readings of peer-reviewed literature on equity in STEM, invited DEI speakers, and/or brainstorming sessions to develop antiracist lab policies. PIs should initiate conversations and actively moderate them to ensure that privileged individuals do not dominate the conversation and racial and ethnic minorities are heard [20].

## Rule 2. Address racism in your lab and field safety guidelines

Racist violence targets BIPOC in the workplace and threatens the ability of students and staff to work safely. Black and Brown people are particularly targeted, even in academic institutions and at research sites. Lab and field safety guidelines should be written with the recognition that some lab members require additional supports to safely conduct their work. Ask BIPOC lab members what you can do to facilitate their safety on campus and in the field. PIs should advocate for BIPOC lab members who may be harassed or harmed by campus security or others that think they don’t “belong” in academic spaces. In the field, PIs should familiarize themselves with any historical and contemporary racist climate present at field sites and prepare accordingly. Provide BIPOC with safety nets such as easy-to-see identification, official-looking field apparel, or work buddies. An open dialogue about race will encourage BIPOC lab members to speak up about what measures they want or need to ensure their safety.

## Rule 3: Publish papers and write grants with BIPOC colleagues

The most important metrics of success in the academy are papers and grants. Publications and grants are also key to tenure, promotion, and career longevity in the academic and other STEM professions. More and more, the most impactful science is done in teams [21], but collaboration networks can be insular. Supportive peer networks in STEM that involve diverse voices produce better quality and highly cited publications [22]. Hence, the most important thing anyone can do to improve the success and retention of BIPOC folx in STEM is to provide opportunities for collaborations that lead to publications and grants. For scientists that work with minoritized communities, it is particularly important to ensure BIPOC are involved in not just manual work and/or data entry but are also provided opportunities to make intellectual contributions that lead to publications and further funding. When organizing workshops or symposia, invite BIPOC scientists to co-lead and not just participate.

## Rule 4: Evaluate your lab’s mentoring practices

Building antiracism into your lab’s mentoring strategy begins with the recognition that racial biases, conscious and unconscious, have the potential to taint mentor–mentee relationships and hinder mentee success. BIPOC mentees report racially motivated gatekeeping behaviors by mentors such as being advised not to pursue advanced degrees or prestigious opportunities, receiving little mentorship in areas associated with issues of identity, experiencing tone policing, and being advised to avoid politics (real and perceived) [23]. Increasingly in STEM, multi-mentor models are being favored over one-on-one top-down mentoring relationships to better center mentee needs and career goals [24]. PIs can help mentees build networks of mentors outside of the lab or institution through culturally relevant professional societies (e.g., Society for Advancement of Chicanos/Hispanics and Native Americans in Science, the different national associations and societies for Black scholars, and Black British Professionals in STEM) or strong online communities (e.g., #BlackandSTEM, #VanguardSTEM, #NativeandSTEM, and #LatinxandSTEM on Twitter) that contribute to a greater sense of agency and confidence and lead to increased academic success. This also encourages lab members to think deeply about their various mentoring needs (e.g., substantive feedback, sponsorship, professional development, and emotional support) and take an active role in cultivating their own science networks [25, 26].

## Rule 5: Amplify voices of BIPOC scientists in your field

Read papers by BIPOC scientists in your lab group meetings, cite the work of BIPOC scholars, and nominate BIPOC for awards. Social media outlets like Twitter are a good place to identify BIPOC scholars that are in your field but outside of your professional bubble. When amplifying BIPOC voices, ensure to highlight their science achievements and not just their contributions to DEI. For example, if you want a BIPOC scientist to speak about DEI to your group or department, first make sure they have a venue to speak about their science. Even if you have a policy of not compensating speakers for presenting on their scholarly work, consider compensating them for the extra labor of educating your community on DEI initiatives.

## Rule 6: Support BIPOC in their efforts to organize

Support the development of safe and brave spaces for BIPOC to organize and discuss issues surrounding race in the absence of white people. For students and trainees, provide meeting space (and additional resources to facilitate effective discussions) where BIPOC can meet and share experiences without fear of retribution. Likewise, support faculty of color in efforts to form separate identity affinity groups within your institution and/or professional organizations. As scholars, we should not forget that our job literally is to educate and mentor the next generation of scholars on how to identify barriers that affect our academic endeavors (including issues related to justice, equity, and inclusion) and come up with plans of actions needed to break down barriers that can prevent us from furthering scientific knowledge.

## Rule 7: Intentionally recruit BIPOC students and staff

After working to foster an inclusive, antiracist lab environment, PIs can begin to evaluate their lab hiring practices for racial or ethnic biases. This rule focusing on increasing lab diversity is purposefully placed after the above rules, which prioritize efforts towards inclusion and retention that should be addressed first. Developing programs for support and retention first helps ensure that BIPOC are not recruited into toxic environments. In recruitment efforts, do not assume racial or ethnic identity from appearances or names; information should be collected from lab members or applicants in a self-reported and voluntary manner. Many of the same efforts used to improve equity in faculty hiring, such as candidate and job ad diversity statements, targeted recruitment of promising candidates, and targeted listservs and databases (e.g., DiverseScholar.org), also apply to recruiting BIPOC lab personnel and trainees [27]. Along with recruiting, PIs can advocate for targeted retention and inclusion initiatives at the department or university level, such as a cohort or cluster approach to diversify STEM student and/or faculty hiring [28, 29].

## Rule 8: Adopt a dynamic research agenda

PIs may be hesitant to hire prospective BIPOC trainees or staff if their research interests do not align closely with the specific research agenda of the lab. A flexible research agenda that accommodates intellectual perspectives outside of the prevailing conversations in one’s field could not only help diversify the lab but also lead to more innovative science. URM scholars produce higher rates of scientific novelty but are also more likely to have their novel contributions discounted and not incorporated into dominant paradigms [30]. PIs, by cultivating dynamic research agendas, can amplify and champion out-of-the-box, innovative contributions from BIPOC scholars.

## Rule 9: Advocate for racially diverse leadership in science

Too often, BIPOC are encouraged to participate in the scientific endeavor in purely supportive or subjugative roles. Go beyond mentoring BIPOC scholars in your lab to sponsoring them (i.e., talk about them to others) to improve their chances of securing jobs, fellowships, awards, and, eventually, leadership roles. Efforts to promote racial and ethnic diversity in science must advocate for BIPOC in leadership positions in labs, institutions, professional societies, editorial boards, and funding agencies. Nominate BIPOC for status elevating roles in science. Early career scholars working in your group should feel empowered to get involved in leadership and advocacy groups within the university and beyond. If possible, their labor in advocating for leadership that addresses the needs of all members of the research group should be accommodated, including with provision of time and resources.

## Rule 10: Hold the powerful accountable and don’t expect gratitude

The goal of cultivating an antiracist lab group is to improve a broader system with known racial inequities. Recognize that white scientists are frequently lauded for DEI work while BIPOC are punished for it [31]. Recognize the difference between performative action and action that doesn’t bring personal glory. We should educate ourselves on effective bystander intervention techniques for addressing issues of inequity, harassment, and discrimination. We should also be able to use accountability mechanisms in our own institutions (if we don’t have them, work to set them up) and hold our colleagues and ourselves accountable for creating healthy workplace climates. Academics are noted for holding those who mishandle text or data (plagiarize or fabricate data) accountable as we consider these acts to be scientific misconduct. Well, if these constitute misconduct, then mistreating people who do the research should definitely rise to the same level of concern and be considered scientific misconduct too [32].

## Conclusion

Scientific labs play an important role in confronting the racism that permeates our social institutions and PIs are uniquely positioned to step up and be leaders in confronting this racism in our everyday work environments. Despite the title of this paper, it may not be easy to rectify the long history of racist behaviors and structures that permeate all scientific disciplines [33]. However, as leaders in science, it is our responsibility to take action and simple concrete steps can and must be made toward addressing individual, institutional, and systemic racism. The work in our labs can begin today; no additional committees, focus groups, or surveys are required.
